# Retarded Learning in a Rabbit Model of Metabolic Syndrome Created by Long-Term Feeding of High-Fat Diet and High Sucrose

**DOI:** 10.3390/nu17193143

**Published:** 2025-10-01

**Authors:** Desheng Wang, Ezekiel A. Irewole, Logan D. Bays, MacKinzie D. Smith, Delanie Talkington, Roger W. Bell, Neha Lal, Bernard G. Schreurs

**Affiliations:** Medical Center Drive, Department of Neuroscience, Rockefeller Neuroscience Institute, West Virginia University School of Medicine, Morgantown, WV 26506, USA; eai00003@mix.wvu.edu (E.A.I.); ldbays@mix.wvu.edu (L.D.B.); mismith1@mix.wvu.edu (M.D.S.); det00006@mix.wvu.edu (D.T.); rbell@hsc.wvu.edu (R.W.B.); neha.lal@hsc.wvu.edu (N.L.)

**Keywords:** high-fat diet, high sucrose, learning, eyeblink conditioning, metabolic syndrome, rabbit, sex difference

## Abstract

**Background**: Metabolic syndrome is a constellation of medical conditions including central obesity, high blood sugar, and high triglycerides known to increase the risk of heart disease, stroke, and type 2 diabetes, with significant sex differences in the syndrome’s incidence and prevalence. These clinical symptoms may be accompanied by cognitive impairment. **Methods:** In the present experiment, we fed rabbits a diet high in fat and sugar (HFSD), assessed symptoms, and measured changes in cognition using trace eyeblink conditioning. **Results:** We show that a range of symptoms of metabolic syndrome resulted from HFSD in male and female rabbits and obesity, high blood sugar, and glucose intolerance were higher in male than female rabbits. Specifically, HFSD male rabbits gained more weight and had a higher body-mass index, more body fat, higher fasting glucose levels, and greater glucose intolerance. Importantly, using trace and delay eyeblink conditioning, we show that there was cognitive impairment because of the high-fat and high-sugar diet in both male and female rabbits, but this was greater in HFSD male rabbits than HFSD female rabbits. **Conclusions:** Metabolic syndrome modeled in rabbits fed a diet high in fat and sugar reflects trends in the adult population including central obesity, high blood sugar, and high triglycerides and cognitive impairment and provides an important model and test bed for assessing interventions.

## 1. Introduction

Clinical metabolic syndrome [1,2,3], defined by the co-occurrence of risk factors such as obesity, high blood sugar, high levels of either cholesterol or triglyceride, and high blood pressure, has been a major public health concern due to the increased risk of cardiovascular disease [4,5], stroke [6], and diabetes [7]. Recent studies [8,9] have pointed to a relationship between metabolic syndrome and neurocognitive decline including an increased risk for dementia. Though the precise mechanisms remain unclear, multiple studies have shown that an earlier onset, a prolonged duration of metabolic syndrome [9,10], and a greater number of metabolic syndrome components [11,12,13] can lead to more severe cognitive deficits later in life [9,10,11,12].

Emerging data [9,14,15,16] also show that these outcomes are not uniform across sexes, suggesting that sex may influence the development, progression, and consequences of metabolic dysfunction, with evidence pointing to sex-specific vulnerabilities in both metabolic regulation and brain function. For example, the overall incidence of metabolic syndrome is higher in men than in women while its prevalence increases with age in women but not in men [17,18], and metabolic syndrome-related organ damage and dysfunction [16,19,20] are more pronounced in women than men. The prevalence and incidence of Alzheimer’s disease are higher in women than in men [21], and metabolic syndrome is significantly associated with cognitive impairment in women [14]. These findings emphasize the need to examine biological sex not as a covariate, but as a central factor in translational models of metabolic syndrome and cognitive impairment.

A high-fat diet (HFD) and high sucrose have been used to mimic clinical metabolic syndrome in rabbits [22,23]. Different groups have manipulated the components of the HFD and the doses of sucrose to mimic different components of metabolic syndrome in rabbits and explore their different research interests, including cardiovascular disease [24,25], liver function [26], renal function [24,25], hypogonadotropic hypogonadism and erectile dysfunction [27,28], sperm alteration [29], retina microaneurysm [23], macular degeneration [30], and carpal tunnel syndrome [31]. However, few studies have addressed how sex modulates these effects, and no work has directly compared males and females in a metabolic syndrome model.

We hypothesized that HFSD would induce key features of metabolic syndrome and impair trace eyeblink conditioning. By explicitly examining sex as a biological variable, our study aims to determine how diet-induced metabolic dysfunction interacts with sex to shape cognitive outcomes, and to establish a preclinical framework for studying sex-specific mechanisms linking metabolism and learning.

## 2. Materials and Methods

### 2.1. Animals

Male and female New Zealand White rabbits (*Oryctolagus cuniculus*) at 6–7 months of age were purchased from Envigo (Indianapolis, IN, USA). The rabbits were housed in individual cages upon arrival, given free access to Purina 5321 rabbit chow and water, and maintained on a 12 h light/12 h dark cycle. After one week of acclimation, rabbits were assigned to one of four groups consisting of males fed normal chow and regular water (Control Male, n = 7), females fed normal chow and regular water (Control Female, n = 7), males fed a high-fat diet and 15% sucrose water (HFSD Male, n = 8), and females fed a HFD and 15% sucrose water (HFSD Female, n = 8).

HFD-fed rabbits received 10% lard incorporated into Purina rabbit chow (TestDiet, Richmond, IN, USA) (Supplementary HFD product sheet) for twenty-eight weeks prior to and throughout the behavioral experiments. Normal chow control rabbits received standard Purina rabbit chow (0% lard) (Supplementary Control diet product sheet) throughout the behavioral experiments. All the experiments complied with National Institutes of Health guidelines for the care and use of animals, and the research was approved by the West Virginia University Animal Care and Use Committee.

### 2.2. Apparatus and NMR Procedures for Behavioral Experiments

The apparatus and recording procedures for the rabbit nictitating membrane response (NMR) have been detailed by Schreurs and Alkon [32], who modeled the apparatus after Gormezano [33]. Specifically, each rabbit was restrained in a Plexiglas box and trained in a sound-attenuating, ventilated chamber (Coulbourn Instruments, Allentown, PA, USA; Model El0-20). A stimulus panel containing a speaker and a house light (10 W, 120 V) was mounted at a 45° angle, 15 cm anterior to and 15 cm above the subject’s head. An ambient noise level of 65 dB was provided by an exhaust fan. A programmable air pressure delivery system (Model ER-3000, Tescom Corp., Elk River, MN, USA) was used to deliver a puff of air through a tube (1 mm internal diameter) positioned 5 mm from and perpendicular to the center of the cornea. Details of transducing NM movements have been reported previously [32,33]. Briefly, a 1 mm hook connected to an L-shaped lever containing a freely moving ball-and-socket joint was attached to a 6-0 nylon loop sutured into, but not through, the NM. The other end of the lever was attached to a potentiometer (Novotechnik US Inc., Southborough, MA, USA; Model P2201) that, in turn, was connected to a 12-bit analog-to-digital converter (5 ms sampling rate; 0.05 mm resolution). Individual analog-to-digital outputs were stored on a trial-by-trial basis for subsequent analysis. Data collection, analysis, and stimulus delivery were accomplished using a LabVIEW (v 5.1) system (National Instruments, Austin, TX, USA).

Rabbits received one day of adaptation, 28 daily sessions of tone and air puff trace conditioning to assess their ability to learn a difficult task, a rest day, 8 daily sessions of tone and air puff delay conditioning to test their ability to learn a simpler hippocampally independent task, and then two daily sessions of tone intensity testing to assess their hearing.

The adaptation was designed to habituate the rabbits to restraint and the training chambers for subsequent daily 60 min sessions of NM movement recording.

The trace conditioning consisted of daily sessions of 60 presentations of a 100 ms, 1 kHz, 82 dB, tone-conditioned stimulus (CS) that was followed by a 500 ms trace interval, and then a 100 ms, 4 psi air puff US (i.e., 600 ms interstimulus interval). To facilitate the delivery of the air puff US, the eyelids were loosely held open during each session with tailor’s hooks attached to an adjustable elastic strap [34]. Stimulus presentations were delivered, on average, every 60 s (50–70 s range). This provided an opportunity to assess the effects of a high-fat diet and high sucrose on the acquisition of a difficult learning task created by the temporal gap or trace between the tone and air puff [35].

The delay conditioning included daily sessions of 60 presentations of a 400 ms, 1 kHz, 82 dB, tone-conditioned stimulus (CS) that coterminated with a 100 ms, 4 psi air puff US.

The tone intensity testing included daily sessions of the presentation of one of the eight 400 ms tone intensities (55, 60, 65, 70, 75, 80, 85, 90 dB) or a zero intensity (0 dB) that coterminated with 100 ms AP. Each of the tone intensities was presented in a randomized sequence that occurred eight times, with each trial delivered, on average, every 60 s (50–70 s range).

An NM conditioned response (CR) was defined as any extension of the NM exceeding 0.5 mm that was initiated after CS onset but before US onset [36].

At the end of the behavioral experiments, rabbits were deeply anesthetized with a mixture of ketamine (35 mg/kg) and xylazine (5 mg/kg), and then overdosed with Euthasol before transcardial perfusion with Dulbecco’s phosphate buffered saline (D-PBS) containing 100 mg/L calcium chloride anhydrous. Brains were extracted after perfusion, frozen on a liquid nitrogen-cooled surface, and stored at −80 °C until further processing.

### 2.3. Comprehensive Metabolic Panel

A plasma metabolic panel including plasma cholesterol, triglycerides, glucose, lipase, liver enzymes, blood urea nitrogen, creatinine, and electrolytes was assessed monthly. The analyses were performed on an automated chemistry analyzer (Catalyst One Chemistry Analyzer, IDEXX Laboratories, Westbrook, ME, USA) with Chem clip 17 with blood samples collected from the central auricular artery of the rabbit ear. The lipase assay was an immunoassay that was read with an optical reader in the Catalyst One Chemistry Analyzer. The units used to express activity are units per liter (U/L).

The Triglyceride–Glucose (TyG) index, a biomarker for insulin resistance, was calculated using fasting triglyceride and glucose levels with the formula TyG = LN [Triglyceride level (mg/dL) × Glucose (mg/dL)/2].

### 2.4. Intravenous Glucose Tolerance Test (GTT)

At the end of the behavioral experiment, all rabbits were subjected to an intravenous glucose tolerance test. Briefly, a blood sample of 0.6 μL was collected right after overnight fasting to measure baseline glucose levels. Then, rabbits were administered a glucose load at a dose of 0.6 g/kg via the intravenous administration of 50% glucose solution (VetOne Dextrose 50% injection, USP, Boise, ID 83705, USA) through the marginal ear vein. Subsequently, blood samples were collected at different time points (15, 30, 60, 90, 120, 180 min) to monitor the changes in blood glucose level after glucose loading. Blood glucose was analyzed with a portable glucose meter (Pet Control HQ Monitor: RA-1A01). The glucose area undercurve (AUC), an index of whole glucose excursion after glucose load, was calculated based on the GTT results.

### 2.5. Enzyme-Linked Immunosorbent Assay (ELISA)

The leptin level in plasma samples was analyzed with a Rabbit Leptin ELISA Kit (ER11RB) from Thermo Fisher Scientific Inc. (Waltham, MA, USA). The adiponectin level in plasma samples was measured with a Rabbit ADP/Acrp30 Adiponectin ELISA kit (Catalog No: MBS763347) from MyBioSource (San Diego, CA, USA). The assays were performed according to the manufacturer’s assay procedure.

### 2.6. Statistical Analysis

All data are presented as mean ± SE. For behavioral studies, factorial repeated measures analyses of variance (SPSS 26, Chicago, IL, USA) were performed on the data with follow-up analyses with LSD correction for the number of repeated comparisons used to localize significant sources of variation. The significance level was set at *p* < 0.05.

## 3. Results

### 3.1. Weight

**Figure 1:**
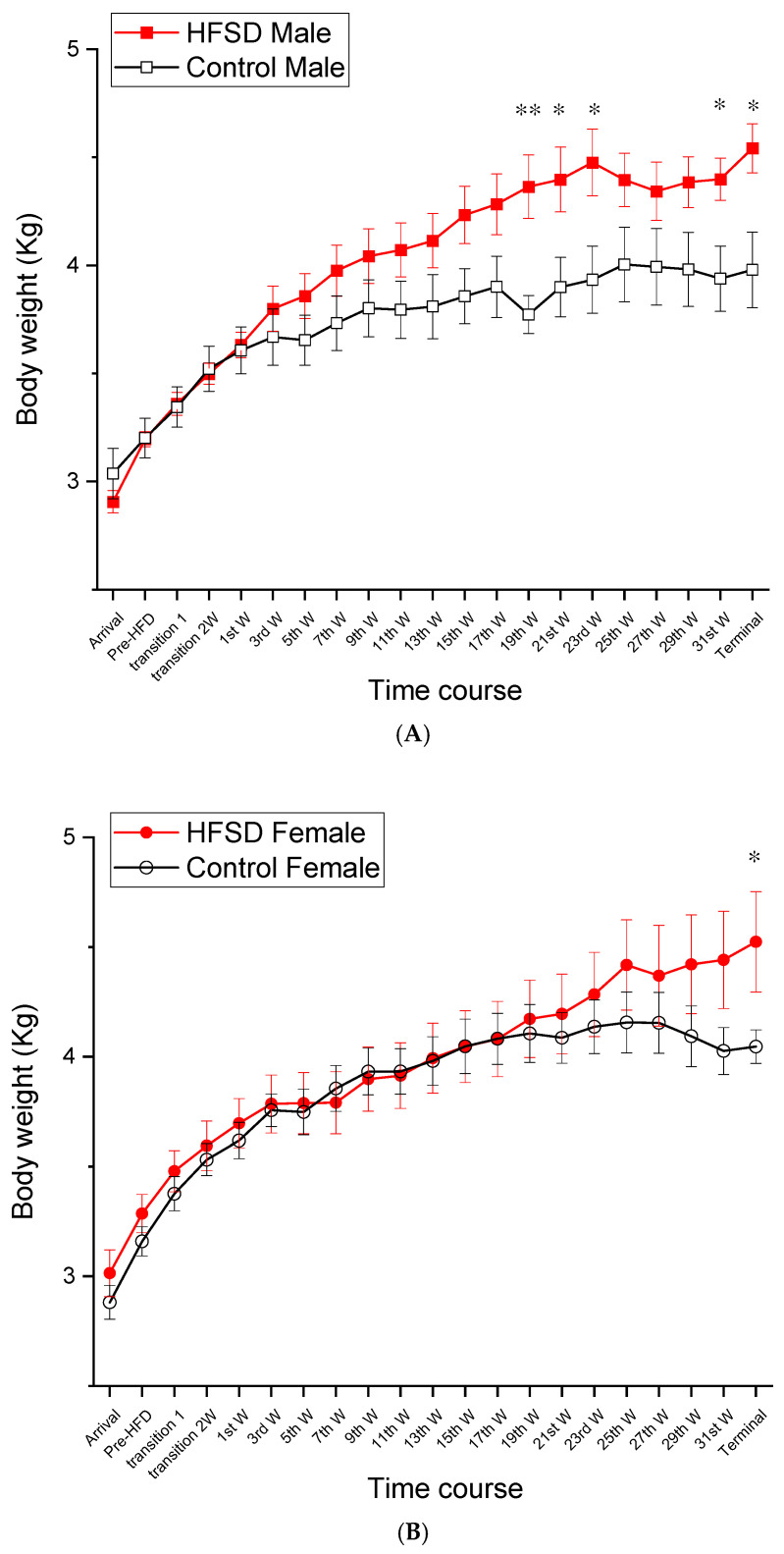
**Rabbit body weight changed as a function of the time duration of the HFSD.** (**A**,**B**) represent the body weights of male rabbits and female rabbits, respectively. The red solid squares and red solid circles are for the HFSD male and HFSD female group, respectively. The black open squares and black open circles are for the Control male and Control female group, respectively. Note that the body weight increased with the time duration of the HFSD, and HFSD rabbits had a relatively bigger body weight than control rabbits, which was prominent in males. This indicated that the HFSD significantly increased body weight when compared to the relative control group. * *p* < 0.05, ** *p* < 0.01. * *p* < 0.05 if compared to relative control. ** *p* < 0.01 if compared to relative control.

Figure 1 shows that there was significant weight gain for all rabbits during the diet duration but that HFSD rabbits had higher weight gains than Control rabbits. Importantly, this occurred earlier and more prominently in HFSD male rabbits than in HFSD female rabbits. These observations were confirmed by two-factor (diet and sex) analysis revealing the effects of diet duration [F(20, 520) = 187.861, *p* = 0.000] and interactions of diet duration × diet [F(20, 520) = 7.745, *p* = 0.000] and diet duration × diet × sex [F(20, 520) = 3.836, *p* = 0.000]. Post hoc comparisons demonstrated that group differences in body weight existed between HFSD males and Control males at diet weeks 19, 21, 23, and 31, and terminally (*p* < 0.01, 0.05, 0.05, 0.05, 0.05, respectively), and between HFSD Sucrose females and Control females terminally (*p* < 0.05). This indicated that a long-term HFSD (10% lard +15% sucrose) did lead to overweight or obesity in rabbits, especially in males.

### 3.2. Lipid Panel Monitoring

**Figure 2:**
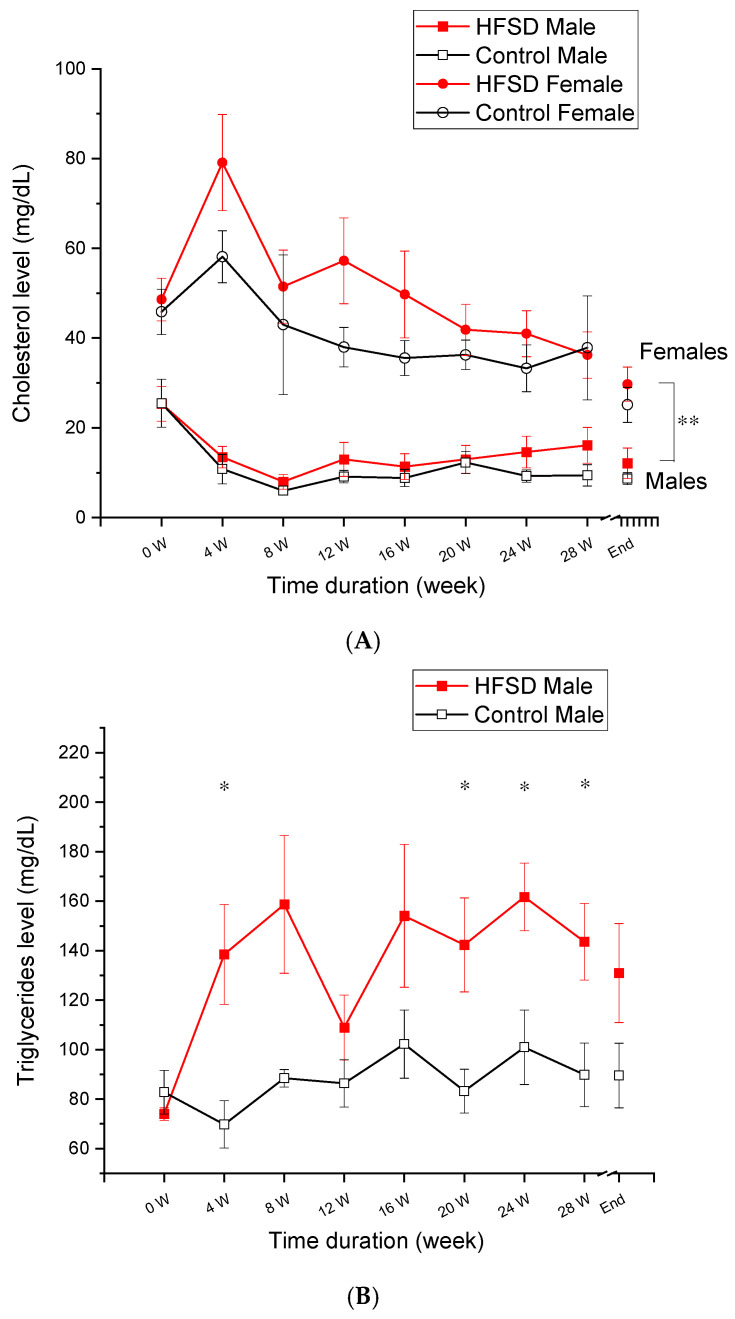

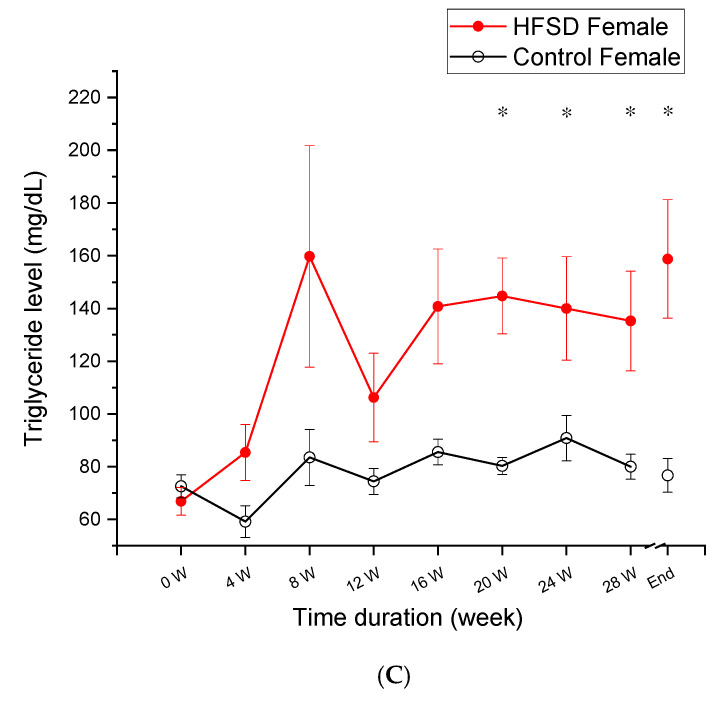
**The lipid panel from rabbits fed with the HFSD or normal chow and regular water.** (**A**–**C**) show that the lipid panel changed as a function of the time duration of the HFSD. The red solid squares and red solid circles represent related value numbers from the HFSD male and HFSD female group, respectively. The black open squares and black open circles represent related value numbers from the Control male group and Control female group, respectively. (**A**) shows that female rabbits had a higher cholesterol level than males, while the HFSD did not affect the cholesterol level if compared to the relative control. (**B**) shows that HFSD male rabbits had a significantly higher triglyceride level than Control male rabbits. (**C**) shows that HFSD female rabbits had a significantly higher triglyceride level than Control female rabbits. In (**A**), ** *p* < 0.01 for comparison between male and female. In (**B**,**C**), * *p* < 0.05 when compared to relative control.

Figure 2 shows that the lipid panel changed as a function of diet duration. As shown in Figure 2A, female rabbits had slightly higher cholesterol levels than males across the duration of the HFSD diet. This was confirmed by two-factor analysis showing a significant sex effect [F(1, 8) = 26.646, *p* < 0.05], but there was no effect of diet [F(1, 8) = 0.278, *p* = 0.612] or interaction of diet × sex [F(1, 8) = 0.002, *p* = 0.963]. Therefore, there was a sex difference in cholesterol level, but the HFSD did not significantly affect cholesterol levels.

Figure 2B,C show that the triglyceride levels changed as a function of HFSD duration in males and females, respectively, with HFSD rabbits having a significantly higher level of triglyceride than Control rabbits. This was confirmed by two-factor analysis showing the main effects of diet [F(1, 8) = 12.555, *p* < 0.01], diet duration [F(8, 64) = 2.951, *p* < 0.01], and an interaction of diet × diet duration [F(8, 64) = 3.119, *p* < 0.01]. However, there was no effect of sex (F’s < 1.9) or any other significant interactions (F’s < 1). Therefore, the HFSD led to dyslipidemia characterized by significantly elevated triglyceride levels in rabbits.

**Figure 3:**
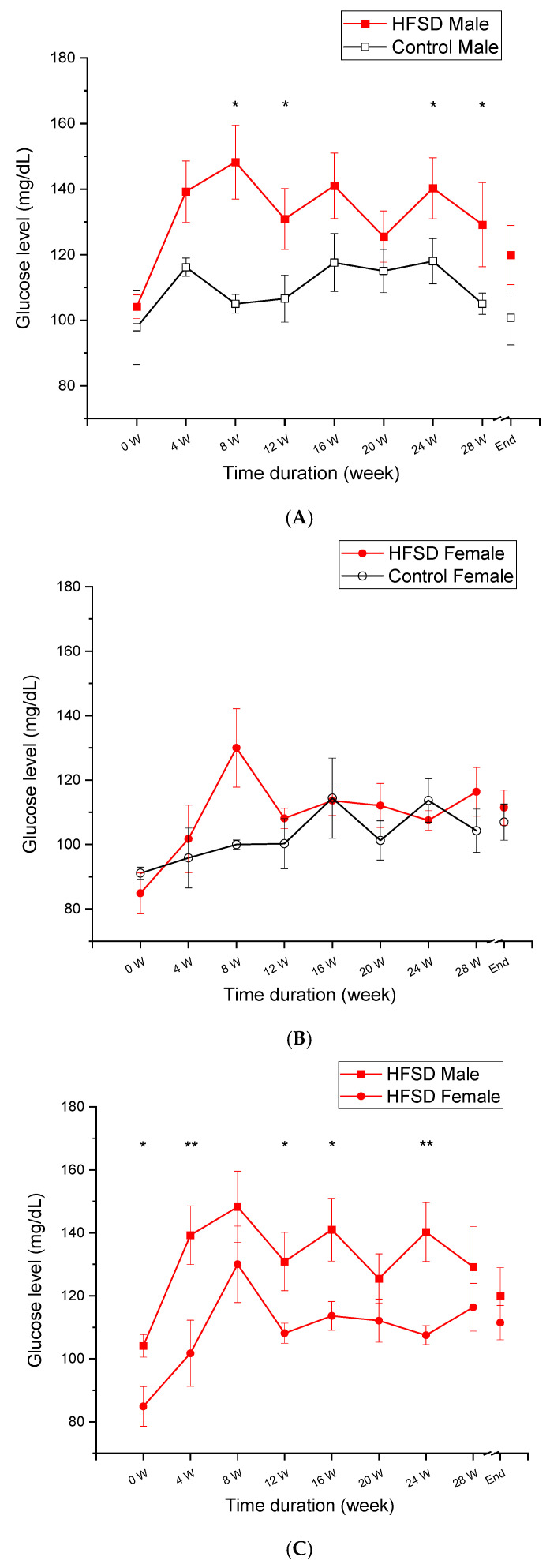
**The HFSD elevated fasting glucose levels.** (**A**–**C**) show that the fasting glucose level changed as a function of the time duration of the HFSD. The red solid squares and red solid circles represent fasting glucose levels in the HFSD male and HFSD female group, respectively. The black open squares and black open circles represent fasting glucose levels in the Control male group and Control female group, respectively. (**A**) shows that HFSD male rabbits had a significant and persistent higher fasting glucose level than control males starting from 4 weeks on the HFSD. This showed that the HFSD significantly affected fasting glucose levels in males. (**B**) shows no significant difference in fasting glucose level between HFSD female rabbits and Control female rabbits. (**C**) shows that HFSD male rabbits had a significantly higher fasting glucose level than HFSD female rabbits, indicating that males may be much more susceptible to the HFSD. * *p* < 0.05, ** *p* < 0.01. In (**A**), * *p* < 0.05 compared to relative Control. In (**C**), * *p*< 0.05, ** *p* < 0.01, when comparing between HFD Sucrose Male and HFD Sucrose Female.

Figure 3 shows that fasting glucose levels changed as a function of the duration of the HFSD. HFSD male rabbits exhibited a significantly higher level of fasting glucose than Control male rabbits (Figure 3A), whereas there was no difference between HFSD female rabbits and Control female rabbits (Figure 3B). Interestingly, HFSD male rabbits also had a higher level of fasting glucose than HFSD female rabbits (Figure 3C). These observations were supported by two-factor analysis revealing the main effects of diet [F(1, 8) = 16.728, *p* < 0.01] and diet duration [F(8, 64) = 7.714, *p* < 0.001], but there were no other significant interaction effects (F’s < 1). Post hoc comparisons showed that the differences were present between HFSD males and Control males at the 8th, 12th, 24th, and 28th weeks (all *p* < 0.05), and between HFSD male rabbits and HFSD female rabbits at weeks 4 (*p* < 0.01), 12 (*p* < 0.05), 16 (*p* < 0.05), and 24 (*p* < 0.01). Thus, the HFSD significantly elevated the fasting glucose level in male rabbits but not female rabbits, suggesting a sex difference in glucose level in response to a long-term HFSD.

### 3.3. Glucose Tolerance Test

**Figure 4:**
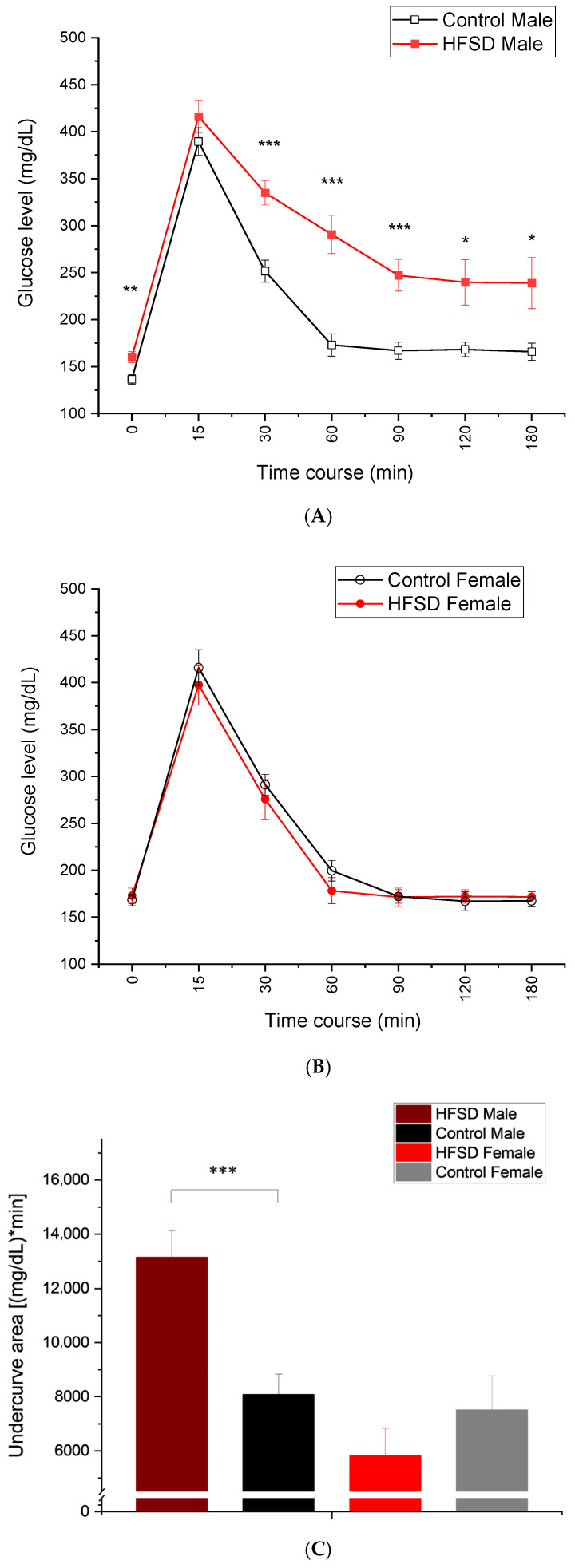
**The HFSD caused glucose intolerance.** (**A**–**C**) show that glucose levels changed during the time course after a glucose load of 0.6 g/kg. Note that the glucose levels spiked at 15 min post glucose load and then gradually decreased during the remaining time course. The red solid squares and red solid circles in (**A**,**B**) represent glucose levels from the HFSD male and HFSD female group, respectively. The black open squares and black open circles represent glucose levels from the Control male group and Control female group, respectively. (**A**) shows that HFSD male rabbits had significantly and persistently higher glucose levels than control males at time points of 60-, 90-, 120-, and 180 min post glucose load. This indicated that the HFSD caused glucose intolerance in males. (**B**) shows no difference in glucose levels between HFSD females and control females at baseline and across all time points post glucose load. This suggested that the HFSD did not cause glucose intolerance in females. (**C**) shows the average undercurve areas for each group, which were calculated based on the glucose levels for each individual rabbit that were collected during glucose tolerance testing. HFSD males had significantly bigger undercurve areas than control males, further confirming the glucose intolerance in males. In (**A**), *, **, and *** correspond to *p* < 0.05, 0.01, and 0.001, respectively, compared to relative Control. In (**C**), *** *p* < 0.001 when comparing between HFD Sucrose males and Control males.

Figure 4 shows that the glucose level changed over time following an intravenous glucose load of 0.6 g/kg. HFSD male rabbits showed elevated baseline glucose levels after overnight fasting compared to Control male rabbits (Figure 4A), while there was no difference in baseline glucose levels between HFSD female rabbits and Control female rabbits (Figure 4B), which was similar to the fasting glucose levels we observed during monthly monitoring. All rabbits demonstrated an immediate and quick increase in glucose levels (glucose spike) at 15 min after a glucose load of 0.6g/kg. However, if compared to control male rabbits, HFSD male rabbits still maintained relatively higher glucose values at 30, 60, 90, 120, and even 180 min post glucose load (Figure 4A), while the glucose levels were very comparable between HFSD female rabbits and Control female rabbits at these time points after glucose load (Figure 4B). These observations were confirmed by two-factor analysis showing the main effect of diet [F(1, 26) = 9.342, *p* = 0.005] and interaction of diet × sex [F(1, 26) = 13.288, *p* = 0.001], though there was no main effect of sex [F(1, 26) = 3.256, *p* = 0.083]. The analysis also showed the effects of the duration post glucose load [F(6, 156) = 327.410, *p* < 0.001] and interactions of duration × diet [F(6, 156) = 2.739, *p* < 0.05], duration × sex [F(6, 156) = 6.584, *p* < 0.001], and duration × diet × sex [F(6, 156) = 4.024, *p* < 0.01]. Significant group differences existed at the time points of 0 min [F(3, 29) = 6.962, *p* < 0.01], 30 min [F(3, 29) = 6.211, *p* < 0.01], 60 min [F(3, 29) = 15.555, *p* < 0.001], 90 min [F(3, 29) = 12.931, *p* < 0.001], 120 min [F(3, 29) = 6.854, *p* < 0.01], and 180 min [F(3, 29) = 6.026, *p* < 0.013], which were attributed to the differences between HFSD males and Control males, and between HFSD males and both HFSD females and Control females. The glucose area under the curve (Figure 4C), an index of whole glucose excursion after glucose load, also showed that HFSD male rabbits had a slower clearance rate. Taken together, the HFSD caused glucose intolerance in males but not females, suggesting a sex difference in rabbits’ metabolic responses to the HFSD.

### 3.4. Triglyceride–Glucose Index

**Figure 5:**
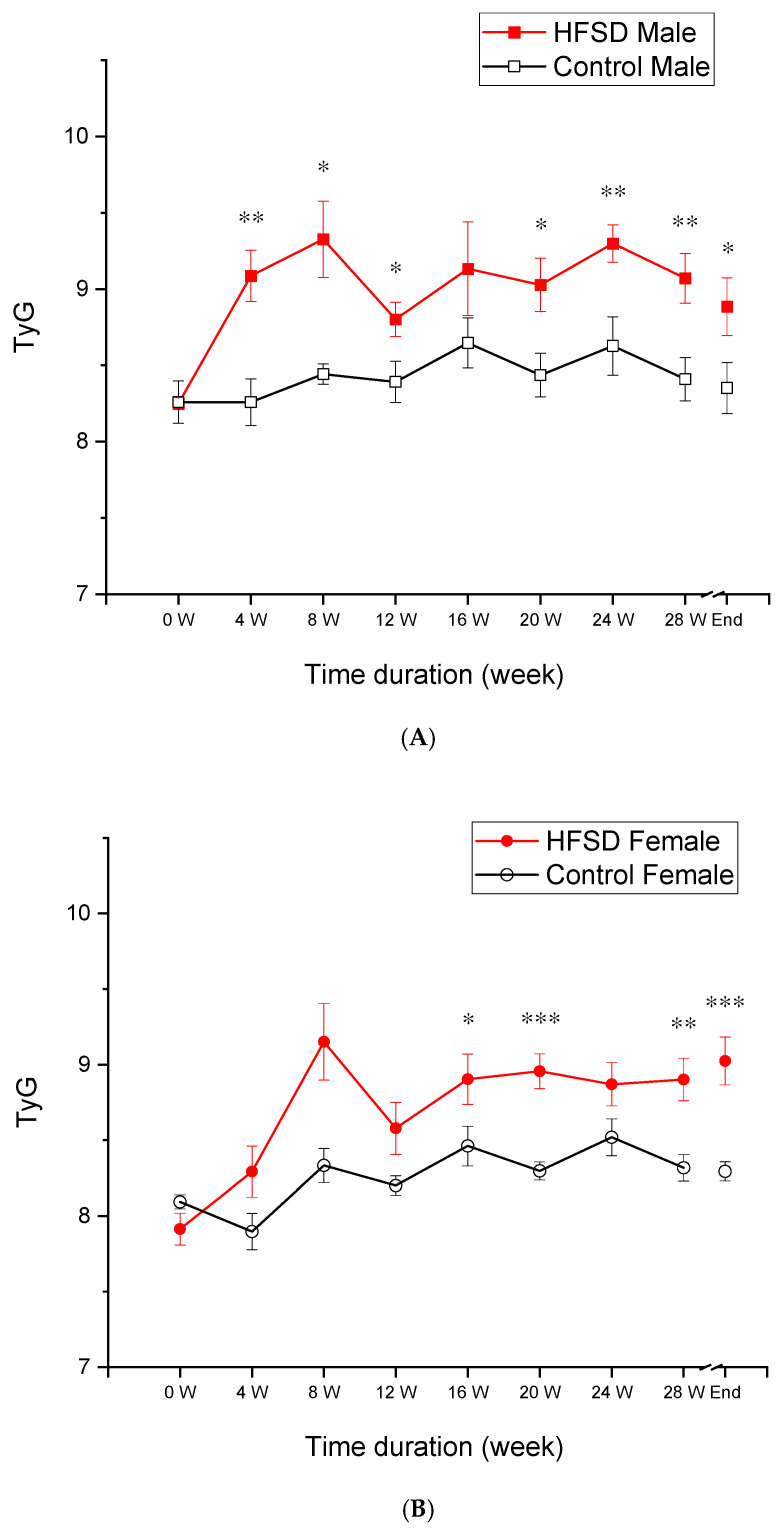
**The HFSD elevated the triglyceride–glucose (TyG) index.** (**A**,**B**) show that the triglyceride–glucose (TyG) index changed as a function of the time duration of the HFSD. The red solid squares and red solid circles in (**A**,**B**) represent the TyG from the HFSD male and HFSD female group, respectively. The black open squares and black open circles represent the TyG from the Control male group and Control female group, respectively. (**A**) shows a significant and persistent increase in the TyG in HFSD males if compared to Control males, which occurred from 4 weeks on the HFSD. This indicated an insulin resistance and prediabetes situation in HFSD males. (**B**) shows a modest and persistent increase in TyG in HFSD females if compared to Control females, which started from 4 weeks on the HFSD. This indicated an insulin resistance and prediabetes situation in HFSD females as well. *, **, and *** correspond to *p* < 0.05, 0.01, and 0.001, respectively, if compared to relative Control.

Figure 5 shows the triglyceride–glucose (TyG) index, which was calculated based on the monthly values for triglyceride and fasting glucose levels, which changed over the duration of the HFSD. If compared to control rabbits, HFSD rabbits had a significantly and persistently higher TyG starting from 4 weeks on the HFSD, which was more prominent in HFSD male rabbits (Figure 5A) than HFSD female rabbits (Figure 5B). These findings were corroborated by two-factor analysis that yielded a main effect of diet [F(1, 8) = 41.016, *p* < 0.001] but marginal sex effect [F(1, 8) = 4.725, *p* = 0.061] and interaction of diet × sex [F(1, 8) = 5.287, *p* = 0.051]. The analysis also showed the effects of diet duration [F(8, 64) = 7.747, *p* < 0.001] and interaction of duration × diet [F(8, 64) = 3.737, *p* < 0.01], but there were no other interactions [F’s < 1]. Post hoc comparisons exhibited the differences in TyG index at almost all time points present between HFSD males and Control males at weeks 4, 8, 12, 20, 24, and 28, and terminally (*p* < 0.01, 0.05, 0.05, 0.05, 0.01, 0.01, and 0.05, respectively), between HFSD females and Control females at weeks 16, 20, and 28, and terminally (*p* < 0.05, 0.001, 0.01, and 0.001, respectively). Therefore, the HFSD did significantly elevate the TyG index in rabbits, suggesting that the HFSD led to insulin resistance and prediabetes in the rabbits.

### 3.5. Terminal Clinical Measures

Table 1 shows clinical measures including weight, length, height, rib cage circumference, length of lower leg, body mass index, and percentage of body fat that were collected right before rabbit euthanasia. Compared to the control rabbits, HFSD rabbits exhibited a larger terminal body weight, rib cage circumference, and body mass index, which were prominent in HFSD male rabbits.

Table 2 lists the weight of total fat tissue, weight of retroperitoneal fat, weight of mesenteric fat, percentage of visceral fat, weight of the liver, weight of the spleen, and weight of the heart in rabbits fed with either the HFSD or control diet. Interestingly, we found significant fat accumulation and increased wet liver weight in HFSD rabbits compared to Control rabbits. ANOVA for terminal clinical measures confirmed significant effects of the HFSD on terminal body weight [F(1, 29) = 12.513, *p* < 0.01], body mass index [F(1, 29) = 10.194, *p* < 0.01], rib cage [F(1, 29) = 13.861, *p* < 0.01], abdominal circumference [F(1, 29) = 8.267, *p* < 0.01], body fat percentile [F(1, 29) = 6.537, *p* < 0.05], total fat [F(1, 29) = 29.816, *p* < 0.001], retroperitoneal fat [F(1, 29) = 23.637, *p* < 0.001], visceral fat [F(1, 29) = 17.661, *p* < 0.001], visceral fat percentile [F(1, 29) = 15.269, *p* < 0.01], and liver wet weight [F(1, 29) = 9.551, *p* < 0.01]. These indicate that the HFSD induced overweight and obesity in rabbits, which was more prominent in male rabbits, suggesting that male rabbits may be more susceptible to the HFSD.

As shown in Table 3, the HFSD might have had a slight effect on blood pressure measures including systolic pressure, diastolic pressure, and MAP in the rabbits, but analysis revealed no statistical difference. Therefore, the HFSD may not model the hypertensive component of clinical metabolic syndrome in our rabbits.

### 3.6. Lipase

**Figure 6:**
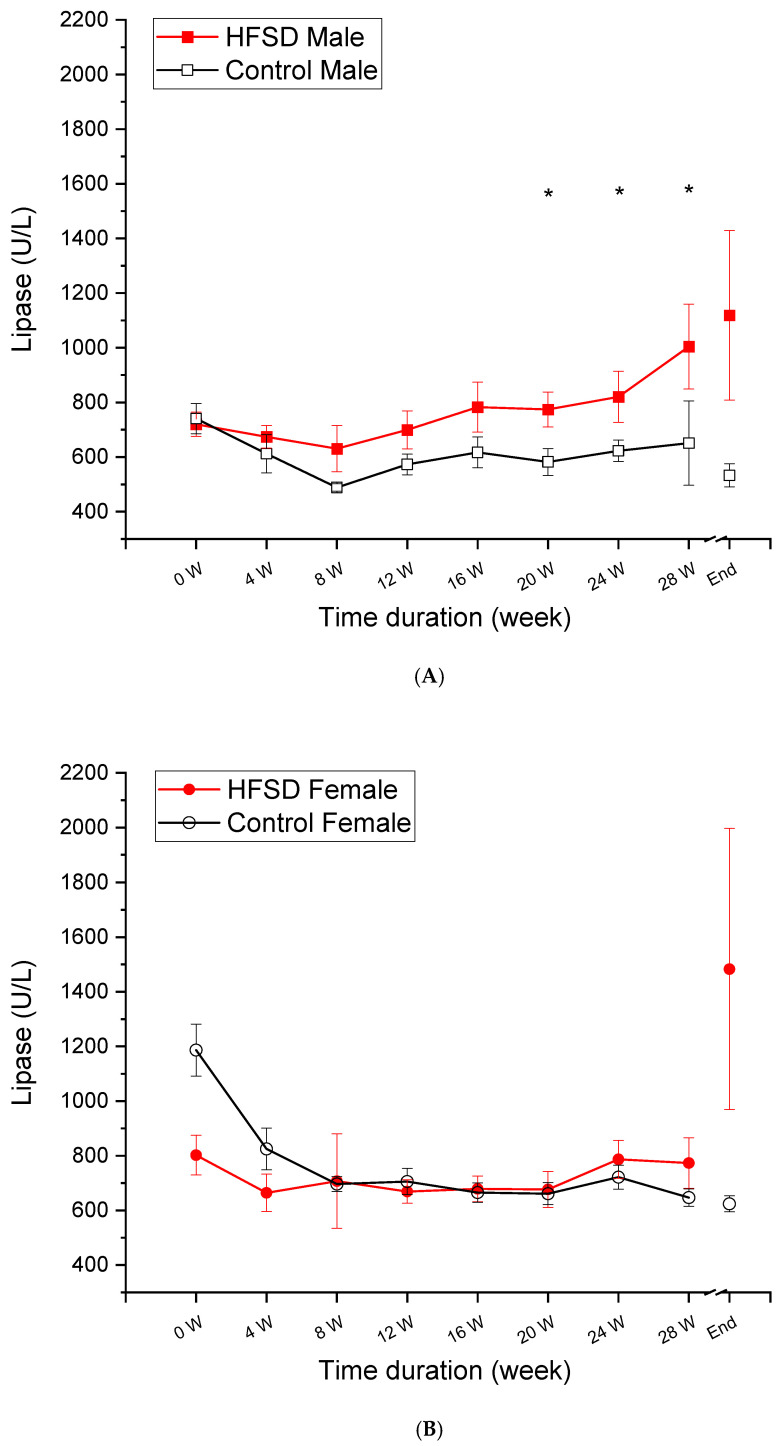
**The HFSD elevated lipase in male rabbits. Figure 6** shows that the lipase changed as a function of the time duration of the HFSD in rabbits. Note that a significant and persistent elevation in lipase was present in males (**A**) across the time duration of the HFSD, but the increase in lipase only occurred at the experiment endpoint in female rabbits (**B**). This indicated that the HFSD may affect pancreas function, especially in males. * *p* < 0.05 if compared to relative Control.

Figure 6 shows that the levels of lipase, a pancreas enzyme, changed as a function of HFSD duration. There was a significant and persistent increase in lipase in HFSD males compared to control males, while the slight elevation in lipase only occurred at the 28th week and terminally in females. The statistical analysis confirmed the effects of diet duration [F(8, 64) = 4.119, *p* < 0.01] and interaction of duration × diet [F(8, 64) = 4.416, *p* < 0.001]. There were no other main effects (F’s <1.6) or interactions (F’s < 1). Post hoc analysis confirmed that the differences present between HFSD males and Control males occurred at weeks 20, 24, and 28 (all *p* < 0.05). Therefore, an HFSD may cause chronic pancreatitis and alter pancreas function in males, which was characterized by chronic and persistent increases in lipase levels and contributed to those alterations in glucose metabolism including elevated fasting glucose, glucose intolerance, and dyslipidemia.

### 3.7. Leptin and Adiponectin Levels

In order to confirm whether the HFSD influenced plasma adipokine levels, ELISA was used to measure leptin and adiponectin levels in plasma samples collected from rabbits at the 28th week of their respective diets. We found that HFSD rabbits had a relatively higher leptin level than Control rabbits [2.74 ± 0.03 ng/mL and 2.63 ± 0.04 ng/mL for HFSD males and Control males, respectively; 2.67 ± 0.05 ng/mL and 2.55 ± 0.05 ng/mL for HFSD females and Control females, respectively]; however, ANOVA revealed only marginal statistical differences in plasma leptin level between HFSD males and Control males (*p* = 0.065), and between HFSD females and Control females (*p* = 0.060).

We also found a slightly higher adiponectin level in HFSD rabbits if compared to Control rabbits [1359.03 ± 141.20 ng/mL and 1087.63 ± 100.68 ng/mL for HFSD males and Control males, respectively; 1184.55 ± 138.58 ng/mL and 1000.97 ± 127.65 ng/mL for HFSD females and Control females, respectively]; however, ANOVA revealed no statistical differences in plasma adiponectin level between HFSD males and Control males (*p* = 0.127), or between HFSD females and Control females (*p* = 0.295).

### 3.8. Trace Conditioning and Delay Conditioning

**Figure 7:**
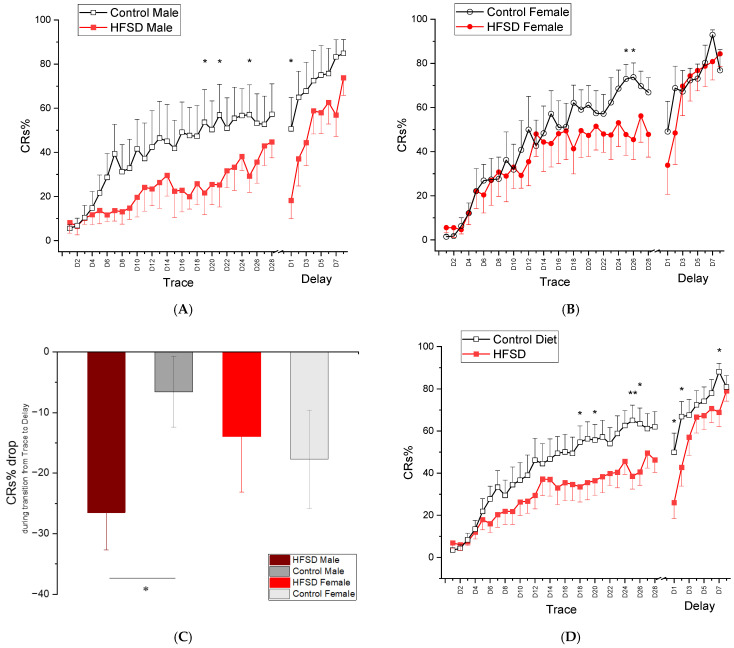
Mean percent (±SEM) conditioned responses (CRs) during 28 days of trace conditioning and 8 days of delay conditioning (**A**,**B**) show the mean percent conditioned responding (CRs%) during 28 daily sessions of trace conditioning and 8 daily sessions of delay conditioning for male rabbits and female rabbits fed with either the HFSD or normal chow and regular water, respectively. The red solid and black open squares in (**A**) represent the CRs% from HFSD male rabbits and Control male rabbits, respectively. The red solid and black open circles in (**B**) represent the CRs% from HFSD female rabbits and Control female rabbits, respectively. Note that the CRs% for both male rabbits and female rabbits increased with training sessions, indicating that all rabbits were able to learn trace conditioning and the HFSD retarded the learning acquisition. (**C**) shows a CRs% drop during transition from trace conditioning to delay conditioning. Note that a significant difference in CRs% drop was present between HFSD males and control males. (**D**) shows the mean percent conditioned responding (CRs%) during 28 daily sessions of trace conditioning and 8 daily sessions of delay conditioning for HFSD rabbits and control rabbits, respectively. HFSD rabbits had a relatively lower CRs% across the sessions of trace conditioning and early sessions of delay conditioning, indicating the retarding effect of the HFSD on trace learning and transition from trace conditioning to delay conditioning. The trace conditioning consisted of twelve 60-trial sessions in which a 100 ms, 82 dB, 1 kHz tone-conditioned stimulus (CS) was presented 500 ms before a 100 ms 4 psi corneal air puff (AP) unconditioned stimulus. The delay conditioning comprised 60-trial sessions in which a 400 ms, 82 dB, 1 kHz tone-conditioned stimulus (CS) was coterminated with a 100 ms 4 psi corneal air puff (AP) unconditioned stimulus. * *p* < 0.05, ** *p* < 0.01.

Figure 7 illustrates the conditioned response percentage (CRs%) of rabbits during both trace conditioning and delay conditioning. All rabbits showed an increase in CRs% during 28 daily sessions of trace conditioning (Figure 7A and Figure 7B for males and females, respectively), and HFSD rabbits had a slower acquisition rate compared to control rabbits (Figure 7A,B,D), suggesting that all rabbits were able to learn a difficult task, a hippocampus-dependent trace conditioning, but it took relatively longer for HFSD rabbits to show CR acquisition. This was confirmed by the significant main effect of session [F(27, 702) = 27.782, *p* < 0.001] and interaction of session × diet [F(27, 702) = 1.626, *p* < 0.05], though there were no interaction effects of session × sex [F(27, 702) = 1.409, *p* = 0.083] or session × diet × sex [F(27, 702) = 0.843, *p* = 0.696].

When shifted to delay conditioning, HFSD rabbits continued to show lower levels of responding at delay day(s) 1–2, but they exhibited rapid acquisition of CRs during the remaining sessions of delay conditioning, and all groups reached similar response levels by the end of delay conditioning. These observations were supported by statistical analysis showing a significant main effect of session [F(7, 182) = 27.646, *p* < 0.001] and interaction of session × diet [F(7, 182) = 2.663, *p* < 0.05]. There were no other main effects or interactions. Note that, during the transition from the last session of trace conditioning to the first session of delay conditioning, HFSD male rabbits exhibited a significant decrease in CRs% compared to control males (Figure 7C). This was confirmed by ANOVA showing a CRs% drop during the transition between HFSD males and Control males (*p* < 0.05).

Taken together, these data indicate that rabbits were able to learn trace conditioning, but the HFSD retarded the acquisition rate in both males and females. The HFSD also made the transition from trace conditioning to delay conditioning much more difficult in males, although all rabbits were able to eventually learn delay conditioning, a simple and cerebellum-dependent task.

### 3.9. Tone Intensity Testing

**Figure 8:**
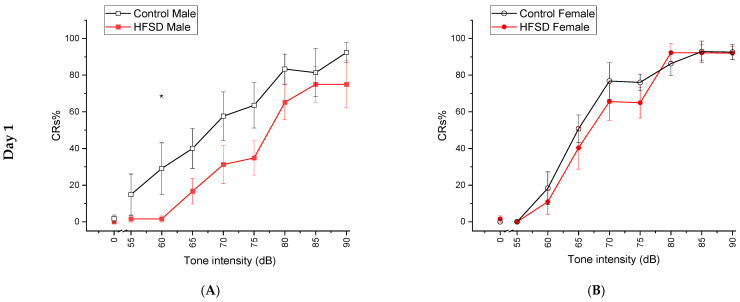

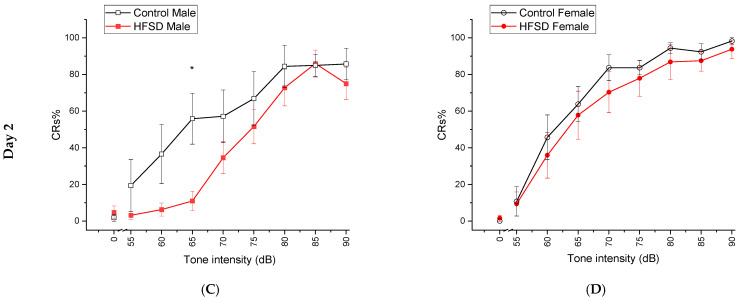
**The HFSD affected the tone threshold at a relatively lower tone intensity but not at the conditioning intensity.** The HFSD affected the tone sensitivity in rabbits. Figure 8 shows the mean percent (±SEM) conditioned responses (CRs) elicited by eight tone intensities (55, 60, 65, 70, 75, 80, 85, 90 dB) or 0 dB in rabbits on day 1 (Top panel) and day 2 (bottom panel) of tone intensity testing, which included 72 trials in total, and each trial consisted of the presentation of a tone with one of those eight intensities that coterminated with corneal air puff. The red solid squares and red solid circles represent the HFSD male (**A**,**C**) and HFSD female (**B**,**D**) group, respectively. The black open squares and black open circles represent the Control male group (**A**,**C**) and Control female (**B**,**D**) group, respectively. Note that statistical differences in CRs% were only present between HFSD males and Control males at relatively lower intensities (60 dB on day 1 and 65 dB on day 2 of tone intensity testing) but not at a conditioning intensity of 82 dB, and there was no difference between HFSD females and Control females. * *p* < 0.05.

To explore whether there were HFSD-induced changes in tone sensitivity and whether the sensitivity changes contributed to the related learning differences that we observed in HFSD rabbits, we conducted two daily sessions of tone intensity testing, which were actually two additional days of delay conditioning but with tones of different intensities. We found that the level of CRs% increased as a function of tone intensity, and the HFSD altered the tone threshold at relatively lower intensities (60 dB on day 1 and 65 dB on day 2 of tone intensity testing) in males (Figure 8A,C) but did not significantly affect tone sensitivity in females (Figure 8B,D). These sensitivity changes did not happen at the conditioning intensity of 82 dB used for trace conditioning and delay conditioning. The analysis of the mean percent CRs elicited by these eight tone intensities in rabbits on day 1 yielded a significant effect of tone intensity [F(8, 208) = 135.768, *p* < 0.001] and interaction of tone intensity × diet [F(8, 208) = 2.396, *p* < 0.05] and tone intensity × sex [F(8, 208) = 4.099, *p* < 0.001]. However, there was no interaction of tone intensity × diet × sex [F(8, 208) = 1.365, *p* = 0.214]. Further analysis confirmed that differences existed between HFSD males and Control males, specifically at the lower tone intensity of 60 dB on day 1 (*p* < 0.05) and tone intensity of 65 dB on day 2 of tone intensity testing. The analysis of the mean percent CRs elicited by these eight tone intensities in rabbits on day 2 showed similar results. Therefore, the HFSD may affect tone sensitivity at relatively low tone intensities but not at the conditioning tone intensity of 82 dB, and thus, the altered tone sensitivity induced by HFSD may not be a significant contributor for the retarded learning we observed in HFSD rabbits.

## 4. Discussion

In this study, we have successfully modeled a clinical metabolic syndrome in rabbits by the long-term feeding of an HFSD (10% lard + 15% sucrose in water), which was characterized by (i) overweight and obesity indicated by an increase in body weight, body mass index, and fat accumulation, including mesenteric fat and retroperitoneal fat; (ii) dyslipidemia indicated by persistent and significantly elevated triglyceride levels; (iii) elevated fasting glucose levels and glucose intolerance; and (iv) a higher triglyceride–glucose (TyG) index. At the same time, we noticed a persistent and significant increase in lipase level in HFSD rabbits, which may suggest pancreatic stress and damage—probably chronic pancreatitis induced by a long-term HFSD. Interestingly, there was a sex difference in the components of metabolic syndrome induced by the HFSD, the changes in which were more prominent in male rabbits than female rabbits; for example, weight gain occurred earlier in male rabbits, there was a significantly higher level of fasting glucose and TyG index in male rabbits than female rabbits, and glucose intolerance was observed only in male rabbits but not in female rabbits.

Importantly, we found retarded trace conditioning in this rabbit model of metabolic syndrome created by the long-term feeding of the HFSD and difficulty in transitioning from trace to delay conditioning—an index of cognitive flexibility—predominantly in male rabbits. In addition, it appears that the long-term HFSD altered the tone threshold at relatively lower intensities (60–65 dB) but not at the conditioning intensity of 82 dB, suggesting that these auditory changes may not be a significant contributor for the retarded trace learning in this rabbit model of metabolic syndrome.

To date, there has been only one other paper showing that an HFD (10% saturated fat coconut oil) and high fructose (30% fructose) for 5 months impaired eyeblink conditioning and produced Alzheimer’s-like pathology in aged female rabbits [37]. The present study was designed to investigate whether chronic consumption of a high-fat (10% lard), high-sucrose (15% in drinking water) diet over 28 weeks would differentially impact male and female rabbits in terms of metabolic outcomes and associative learning.

A high-fat diet and high sucrose have been used to model clinical metabolic syndrome in rabbits due to their herbivorous physiology and pronounced sensitivity to dietary fat [22,38,39]. Additionally, rabbits share greater sequence homology with humans in cholesteryl ester transfer protein [40], enhancing their translational relevance for studying human lipid metabolism and metabolic disease. For example, dietary cholesterol and copper-fed rabbits have shown at least 12 pathological markers observed in AD, including increased beta-amyloid accumulation, senile plaque-like structures in the hippocampus and temporal lobe, and significantly retarded trace learning [39]. Our current data from rabbits fed with an HFSD (10% lard +15% sucrose) show clear signs of obesity and central adiposity including increased body weight, elevated BMI, and significant mesenteric fat accumulation. These are consistent with earlier reports showing similar metabolic outcomes in 3-month-old male rabbits fed with an HFD [15% fat (10% corn oil and 5% lard)] for 18 weeks [41] and 4-month old female rabbits fed the same diet for 12 weeks [42]; in 3-month-old male rabbits fed with an HFD [10% lard + (0.5% cholesterol during first 12 weeks then 0.1%) and high sucrose (40% sucrose)] for 24 weeks [23]; and in 4–5-month-old male rabbits fed with an HFD (10% hydrogenated coconut oil + 5% lard) and high sucrose (15% dissolved in water) for 28 weeks [22]. In contrast, no comparable excess weight or obesity in rabbits fed with a mild HFD alone (5% lard + 5% soy oil) was observed [43], suggesting that there may be either dose-dependent effects or synergistic effects of high fat and sucrose in our rabbit model of metabolic syndrome created by an HFSD (10% lard + 15% sucrose).

However, some studies also reported no significant weight changes among male rabbits fed with one of four different types of diet [HFD (10% corn oil + 8% lard), 1% cholesterol diet, HFD (10% corn oil + 8% lard) plus 1% cholesterol, and Control diet] for 6 weeks [44]; lesser weight gain in male rabbits fed with an HFD (10% lard) and high sucrose (37% sucrose) for 6 months [45]; or even weight loss in 3-month-old male rabbits fed an HFD (10% fat) and high sucrose (30% sucrose) for 48–56 weeks [46]. These discrepancies may be attributed to the duration of the diet, components of the diet, and specific types of water [43,44,45,46], and whether there was an impairment in health induced by the unpalatable diet [45,46]. Importantly, in our model, male rabbits exhibited greater weight gain than females despite identical HFSD exposure, reinforcing a sex-dependent metabolic vulnerability.

Our data also demonstrated a persistent elevated triglyceride level in both male and female rabbits fed with the HFSD, which is similar to previous reports in 4–5-month-old male rabbits fed with an HFD (10% hydrogenated coconut oil + 5% lard) and high sucrose (15% dissolved in water) [22]; in male rabbits fed with an HFD (10% lard) and high sucrose (37% sucrose) for 6 months [45]; in 3-month-old male rabbits fed an HFD (10% fat) and high sucrose (30% sucrose) for 48–56 weeks [46]; and in aged female rabbits fed an HFD (10% coconut oil) and high fructose (30% fructose) for 4 months [37]. Notably, elevated triglycerides were present regardless of either weight gain [23] or lesser weight gain or even weight loss [45,46]. This suggests that triglycerides serve as an independent and reliable metabolic index for the effects of an HFSD. However, triglyceride levels remained unchanged in rabbits fed with an HFD (5% lard + 5% soy oil) for 20 weeks [43] and HFD [15% fat (10% corn oil and 5% lard)] for 18 weeks [41], indicating that it may depend on the components of the HFD and dose of sucrose.

Our data show no HFSD-induced changes in total cholesterol levels, which is similar to previous reports in rabbits fed with an HFD (5% lard + 5% soy oil) for 20 weeks [43]; HFD [15% fat (10% corn oil and 5% lard)] for 12 weeks [42] and for 18 weeks [41]; HFD (10% corn oil + 8% lard) for 6 weeks [44]; HFD (10% hydrogenated coconut oil + 5% lard) and high sucrose (15% dissolved in water) for 28 weeks [22]; HFD (10% fat) and high sucrose (30% sucrose) for 48–56 weeks [46]; and HFD (10% coconut oil) and high fructose (30% fructose) for 4 months [37]. Therefore, a typical HFSD may not reliably affect cholesterol levels unless cholesterol is directly supplemented in the diet [23,37,44].

Our data showed that HFSD male rabbits developed persistently and significantly elevated fasting glucose levels, glucose intolerance, and a higher TyG index, indicating insulin resistance, the impairment of acute and chronic insulin responses to glucose load, and a prediabetes state. Interestingly, we found higher fasting glucose levels and a higher TyG index in HFSD males than HFSD females, and we did not observe HFSD-induced glucose intolerance in female rabbits, indicating a clear sex difference in glucose metabolism following HFSD exposure. These results are in accordance with previous findings of glucose impairment in male rabbits fed with an HFD [15% fat (10% corn oil and 5% lard)] for 18 weeks [41]; HFD (10% coconut oil) for 22 weeks [47]; and HFD (10% hydrogenated coconut oil + 5% lard) and high sucrose (15% dissolved in water) for 28 weeks [22]. Conversely, no abnormal glucose metabolism was triggered in male rabbits fed with an HFD (3% coconut oil) for 22 weeks [47] and HFD (5% lard + 5 soy oil) for 20 weeks [43]. We did not find the same changes in fasting glucose levels and glucose intolerance in HFSD female rabbits as prior reports in 4-month-old female rabbits fed with an HFD [15% fat (10% corn oil and 5% lard)] for 12 weeks [42] and in 24–32-month-old female rabbits fed with an HFD (10% coconut oil) and high fructose (30%) [37]. We found a higher TyG index, which is indirect evidence for insulin resistance and prediabetes, predominantly in HFSD male rabbits, indicating that the HFSD induced a prediabetes condition and insulin resistance, which is in line with previous studies in male rabbits fed with an HFD (10% coconut oil) for 22 weeks [47] and in female rabbits fed with an HFD [15% fat (10% corn oil and 5% lard)] for 12 weeks [42]. Therefore, abnormal glucose metabolism may be one of the most important and reliable metabolic indexes in our rabbit model of metabolic syndrome. The sex-biased vulnerability suggests that male rabbits are more susceptible to HFSD-induced glycemic dysregulation, while females may retain partial metabolic protection.

Note that we observed a persistent elevation in circulating lipase levels in HFSD males but not females. This may reflect subclinical or chronic pancreatic injury specific to males, and it could help explain the glucose intolerance and fasting hyperglycemia observed in this group.

Notably, we did not observe elevated blood pressure in HFSD-fed rabbits. This contrasts with other reports of hypertension in adult males fed high-fat diets [22,25,42]. It is worth noting that our blood pressure measurements were taken from sedated rabbits using a rear-leg cuff, rather than through direct monitoring via artery catheterization in conscious animals. As a result, we cannot entirely rule out subtle hemodynamic changes.

Our data show that the HFSD retarded learning in both males and females, with rabbits having a slower acquisition rate during trace eyeblink conditioning and lower levels of responding at day(s) 1–2 during delay eyeblink conditioning, and it made the transition from trace conditioning to delay conditioning much more difficult in males. This has been verified by a separate analysis revealing lower levels of CRs% in HFSD rabbits compared to controls across a number of days, and a significant decrease in CRs% from the last session of trace conditioning to first session of delay conditioning present only in HFSD male rabbits. Since a long-term mild HFD facilitates discrimination learning in rabbits without metabolic syndrome [43], the retarded trace learning in current study is more likely due to specific components of metabolic syndrome induced by the HFSD or a synergistic interaction among them rather than fat exposure alone. Our data align with clinical studies linking metabolic syndrome to dementia risk [9,10,13,48,49]. Our data are also supported by one preclinical study [37] showing that the 4-month feeding of an HFD (10% coconut oil) and high fructose (30% fructose) impairs both spatial memory and trace eyeblink conditioning in aged female rabbits, and by some other studies in rodents showing that HFD-related metabolic syndrome impairs learning and memory [50,51] and that the repeated infusion of plasma collected from age-matched low-fat-fed mice changes the metabolic profile and improves memory in HFD mice [52].

The mechanisms behind these impairments remain incompletely understood. However, elevated triglyceride levels in midlife can predict beta-amyloid and tau pathology 20 years later in cognitively healthy individuals [53]; high triglyceride levels are linked with greater cognitive decline and dementia risk [49,54,55]; high blood glucose levels [56], high-glycemic diets, and insulin resistance [57] are associated with cognitive deficit and Alzheimer’s pathology; and a higher TyG index is correlated with elevated plasma amyloid beta 42 and an increased likelihood of dementia. Therefore, we assume that the retarded trace eyeblink conditioning in HFSD rabbits may be driven by a convergence of metabolic disruptions including elevated triglycerides, abnormal glucose metabolism such as an elevated fasting glucose level and glucose intolerance, a higher TyG index, and overweight and central obesity characterized by increased mesenteric fat mass. These factors may modulate brain function and structure through the regulation of amyloid production [37,58,59], neuronal plasticity [59,60], BDNF signaling [59,61], oxidative stress [59], microglial activation, and neuroinflammation [61,62,63].

An HFD has been regarded as a risk factor for age-related hearing loss. Our data showed that an HFSD affected the tone threshold only in male rabbits at relatively lower tone intensities of 60–65 dB but not at the conditioning tone intensity of 82 dB. This is consistent with clinical findings showing that chronic dyslipidemia and elevated triglycerides lead to reduced auditory function and hearing decline [64,65] and animal studies showing that 60 kcal% fat-diet-induced obesity exacerbates auditory degeneration in CD/1 mice [66] and hearing impairment in CBA/Ca mice [67]. Note that an HFSD does not affect the tone threshold in female rabbits, suggesting that female rabbits may be more resistant to the negative effects of an HFSD including its effects on hearing, body weight, and metabolism, as shown by a previous study in CBA/Ca mice [67].

## 5. Conclusions

This is the first report of sex differences in components of metabolic syndrome and retarded trace eyeblink conditioning in a rabbit model of metabolic syndrome created by feeding an HFSD, which reinforces the importance of the NIH’s research policy on sex as a biological variable in preclinical metabolic research. Our previous data [43] suggest an HFD may have protective effects on behavioral performance, but the present data suggest that, once metabolic syndrome occurs, behavioral performance degrades.

## Figures and Tables

**Table 1:** Clinical measures from rabbits fed with HFSD or normal chow and regular water.

	Weight (kg)	Length (m)	Height (m)	Rib Cage (cm)	Length of Lower Leg (cm)	Abdominal Circumference	BMI (kg/m^2^)	Percentage of Body Fat
HFSD Female	4.53 ± 0.23 ^§^	0.35 ± 0.006	0.157 ± 0.004	34.63 ± 0.79 *	13.88 ± 0.17 *	35.94 ± 1.38	82.29 ± 1.77 *	20.00 ± 1.19
Control Female	4.04 ± 0.07	0.342 ± 0.008	0.163 ± 0.003	32.29 ± 0.49	13.29 ± 0.16	33.07 ± 0.81	72.91 ± 3.00	17.92 ± 0.79
HFSD Male	4.53 ± 0.12 *	0.352 ± 0.006	0.157 ± 0.004	35.50 ± 0.74 *	13.88 ± 0.28	35.5 ± 0.96 *	82.269 ± 2.18 *	21.24 ± 1.11 ^§^
Control Male	3.97 ± 0.18	0.341 ± 0.010	0.153 ± 0.004	32.29 ± 1.14	13.64 ± 0.31	32.57 ± 1.16	76.30 ± 3.56	17.17 ± 1.98

Length is the distance from the point of the shoulder to the tuber ischium; Height is the distance from the point of the shoulder through the point of the elbow; LLM is the length of the lower leg, from the middle of the patella to the dorsal tip of the calcaneal process in centimeters; BMI = Body weight (Kg)/[Body length (m) × Height (m)]; Percentage of body fat = (rib cage/0.7062) − (llm/0.9156) − lim. * *p* < 0.05 if compared to relative Control. ^§^ *p* = 0.06 if compared to relative Control.

**Table 2:** Fat and liver weights from rabbits fed with HFSD or normal chow and regular water.

	Total Fat (g)	Retroperitoneal Fat (g)	Mesenteric Fat (g)	% Visceral Fat	Liver (g)	Spleen (g)	Heart (g)
HFSD Female	602.48 ± 81.24 **	313.12 ± 58.43 *	289.35 ± 40.99 *	6.31 ± 0.77 *	106.89 ± 11.44 *	2.03 ± 0.29	7.49 ± 0.57
Control Female	321.34 ± 18.26	155.56 ± 8.14	165.78 ± 15.07	4.11 ± 0.37	76.35 ± 7.38	1.93 ± 0.39	7.81 ± 0.46
HFSD Male	494.88 ± 33.66 ***	260.91 ± 15.16 ***	233.97 ± 19.27 **	5.14 ± 0.33 **	119.04 ± 8.90 ^§^	1.31 ± 0.10	8.73 ± 0.44
Control Male	264.83 ± 16.39	120.14 ± 3.97	144.69 ± 17.92	3.59 ± 0.26	94.75 ± 8.20	1.09 ± 0.13	8.02 ± 0.24

% Visceral fat = 100 × Mesenteric fat (g)/[body weight (kg) × 1000]; * *p* < 0.05 if compared to relative Control, ** *p* < 0.01 if compared to relative Control, *** *p* < 0.001 if compared to relative Control. ^§^ *p* = 0.053 if compared to relative Control.

**Table 3:** Blood pressure and heart rate from rabbits fed with HFSD or normal chow and regular water.

	SBP (mmHg)	DBP (mmHg)	MAP (mmHg)	Heart Rate (BPM)
HFSD Female	128.94 ± 8.71	75.84 ± 8.17	93.54 ± 8.06	189.19 ± 10.83 *
Control Female	120.03 ± 3.00	59.87 ± 5.82	79.93 ± 4.26	160.54 ± 7.46
HFSD Male	120.96 ± 5.04	66.94 ± 5.54	84.95 ± 5.26	191.2 ± 8.46
Control Male	118.51 ± 3.84	60.24 ± 1.45	79.66 ± 1.78	181.89 ± 8.31

SBP: systolic blood pressure; DBP: diastolic blood pressure; MAP: mean arterial pressure; BPM: beats per minute. MAP = (SBP + 2 × DBP)/3; * *p* < 0.05 if compared to relative Control.

## Data Availability

The raw data supporting the conclusions of this article will be made available by the authors on request.

## Supplementary Materials

The following supporting information can be downloaded at: https://www.mdpi.com/article/10.3390/nu17193143/s1.
